# Telephone‐guided imagery rehearsal therapy for nightmares: Efficacy and mediator of change

**DOI:** 10.1111/jsr.13123

**Published:** 2020-06-21

**Authors:** Jaap Lancee, Marieke Effting, Anna E. Kunze

**Affiliations:** ^1^ Department of Clinical Psychology University of Amsterdam Amsterdam The Netherlands; ^2^ PsyQ Amsterdam Amsterdam The Netherlands; ^3^ Department of Psychology LMU Munich Munich Germany

**Keywords:** imagery rehearsal treatment, mastery, nightmares, self‐help

## Abstract

The currently best‐supported psychological treatment for nightmares is imagery rehearsal therapy. The problem, however, is that not enough trained practitioners are available to offer this treatment. A possible solution is to conduct imagery rehearsal therapy in a guided self‐help format. In the current study, 70 participants with nightmares according to the fifth edition of the Diagnostic and Statistical Manual of Mental Disorders were randomized to either telephone‐guided imagery rehearsal therapy (*n* = 36) or a wait‐list condition (*n* = 34). Participants in the imagery rehearsal therapy condition received three sessions over the course of 5 weeks. Every treatment session was followed by telephone support delivered by postgraduate students. Participants who received imagery rehearsal therapy showed larger improvements on nightmare frequency (*d* = 1.03; *p* < .05), nightmare distress (*d* = 0.75; *p* < .05) and insomnia severity (*d* = 1.12; *p* < .001) compared with the participants in the wait‐list condition. The effects were sustained at 3‐ and 6‐month follow‐up. No significant effects were observed on the number of nights with nightmares per week, anxiety and depression. In line with earlier reports, the treatment effect was mediated by the increase of mastery at mid‐treatment, underlining the mechanistic value of mastery in imagery rehearsal therapy. The present study demonstrates that it is possible to deliver imagery rehearsal therapy in a self‐help format supported by unexperienced therapists and with relatively little time investment. This opens possibilities in terms of cost‐effectiveness, scalability and dissemination of imagery rehearsal therapy in the treatment of nightmares.

## 1 INTRODUCTION

According to the Diagnostic and Statistical Manual of Mental Disorders, Fifth Edition (DSM‐5), nightmares are well remembered, dysphoric dreams that often lead to awakening (American Psychiatric Association, 2013). To fulfil the criteria for nightmare disorder, the nightmares should not be explained by other factors, and they should cause significant clinical distress in daily life. Nightmares can be of idiopathic (with no specific origin) or post‐traumatic nature. Two−five percent of the general population reports one or more nightmares per week (Li, Zhang, Li, & Wing, 2010; Sandman et al., 2013; Schredl, 2010). This number increases to 30% within psychiatric populations (Swart, van Schagen, Lancee, & van den Bout, 2013). Having nightmares is associated with higher levels of distress (Lancee & Schrijnemaekers, 2013) and with psychopathology (van Schagen, Lancee, Swart, Spoormaker, & van den Bout, 2017).

Nightmare sufferers rarely seek help (Gieselmann et al., 2019), even though there are various treatments available for nightmares. The two main treatment options are the alpha‐1 antagonist prazosin and the psychological treatment imagery rehearsal therapy (IRT; Seda, Sanchez‐Ortuno, Welsh, Halbower, & Edinger, 2015; Yücel, van Emmerik, Souama, & Lancee, 2020). The efficacy of prazosin treatment for nightmares has mainly been tested for post‐traumatic nightmares, and has recently been under debate because a large‐scale trial could not detect any relevant treatment effects (Morgenthaler et al., 2018; Raskind et al., 2018). However, a recent meta‐analysis still indicated efficacy for both treatment formats with similar effect sizes (Yücel et al., 2020).

Imagery rehearsal therapy is an effective treatment (range *d* = 0.48–0.55) that has been tested for both post‐traumatic and idiopathic nightmares (Augedal, Hansen, Kronhaug, Harvey, & Pallesen, 2013; Hansen, Hofling, Kroner‐Borowik, Stangier, & Steil, 2013; Yücel et al., 2020). In an earlier study, we demonstrated the efficacy of face‐to‐face IRT delivered in an isolated single‐component treatment format (Kunze, Arntz, Morina, Kindt, & Lancee, 2017).

It is hypothesized that “mastery” is an important treatment mechanism of IRT. In the nightmare literature, mastery is operationalized as the conviction of being in control over one's nightmare (Rousseau & Belleville, 2018). Given that nightmare patients typically experience powerlessness and uncontrollability with regard to their nightmares, IRT might offer a means to express unmet needs and inhibited responses. The expression of such previously inhibited action tendencies, feelings or needs during IRT may (re‐)establish a feeling of mastery of the nightmare content and eventually lead to the reduction of associated symptoms (Kunze, Lancee, Morina, Kindt, & Arntz, 2019). A few studies have indeed shown the relationship between mastery and IRT’s efficacy (Germain et al., 2004; Krakow et al., 2001); however, only one study showed that mastery mediated the effects of IRT (Kunze et al., 2019). Therefore, a replication of this finding is much needed before treatment development directed toward increased mastery is further explored.

Another issue with IRT is the problem of dissemination as there are not enough trained therapists available to deliver this treatment. One solution may be to deliver IRT in a guided self‐help format. In the past decade, several guided internet‐delivered treatments for various psychological problems have emerged with effects comparable to their face‐to‐face counterparts (Carlbring, Andersson, Cuijpers, Riper, & Hedman‐Lagerlof, 2018; Cuijpers, Donker, van Straten, Li, & Andersson, 2010). For IRT, there have only been two controlled studies, with one study demonstrating the effect of IRT in a self‐help booklet (Lancee, Spoormaker, & van den Bout, 2010) and one study in an online format (Gieselmann, Bockermann, Sorbi, & Pietrowsky, 2017).

In order to extend the earlier findings in the current study, we aimed to demonstrate the efficacy of IRT in a telephone‐guided self‐help format with a protocol similar to the isolated treatment as employed in our earlier study (Kunze et al., 2017). Also, we aimed to replicate the finding that mastery is a mediator of the efficacy of IRT (Kunze et al., 2019). We expected the following.

- Telephone‐guided IRT is more effective than a control condition on nightmare frequency, nightmare distress and several secondary outcomes.
- Mastery is a mediator of the effect of IRT on nightmare frequency and nightmare distress.

## 2 MATERIALS AND METHODS

### 2.1 Participants

**FIGURE 1:**
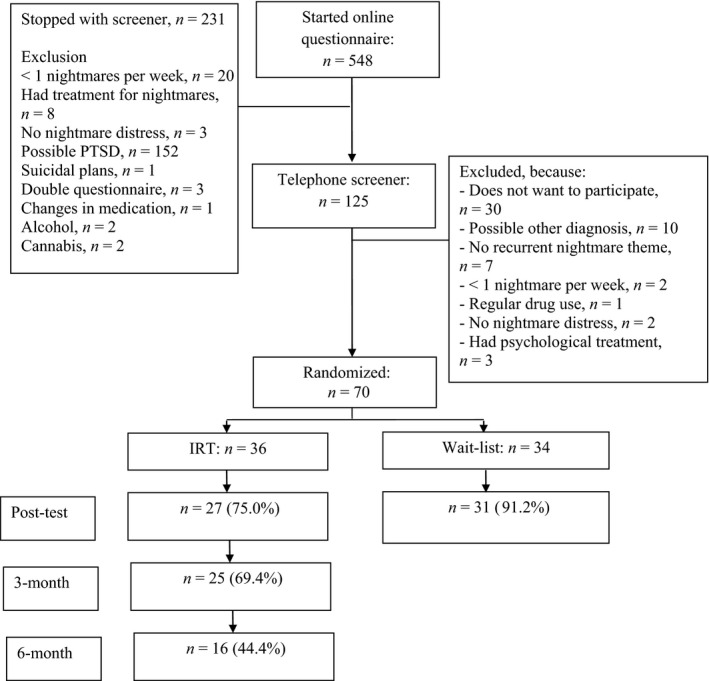
Flowchart

Participants were recruited from March 2018 to November 2018 via a popular‐science website and Facebook advertisements. A total of 548 interested volunteers started the online questionnaire, of which 70 participants were randomized to either the IRT (*n* = 36) or the wait‐list (WL) condition (*n* = 34; see Figure 1 for participant flow). The included sample was aged between 20 and 58 years, predominately female, of higher education, and of Dutch descent (see Table 1 for the sample demographics). Inclusion criteria were: (a) nightmare disorder according to the DSM‐5; (b) at least one recurrent nightmare per week, defined as either replications of a single nightmare or different nightmares following a general theme (e.g. being chased); (c) 18 years or older; (d) access to the internet and a valid telephone number. Exclusion criteria were: (a) psychological treatment for nightmares in the last 12 months; (b) indication for post‐traumatic stress disorder (PTSD) based on the PTSD Symptom Scale (PSS; Foa, Riggs, Dancu, & Rothbaum, 1993); as well as current (c) psychosis/schizophrenia; (d) concrete suicidal plans; (e) alcohol or cannabis abuse; (f) unstable use of medication for psychological complaints (with the exception of incidental use of medication for insomnia). People were not excluded based on other sleep disorders such as sleep apnea or other parasomnias.

**TABLE 1:** Demographic and clinical characteristics at baseline

		IRT	WL	
Age	*M* (*SD*)	29.5 (8.6)	29.7 (10.4)	*F* (_1, 68_) = 0.003, *p* = .96
Gender	Female	97.2% (35)	94.1% (32)	*χ* ^2^(1) = 0.41, *p* = .61
Education	High	72.2% (26)	88.2% (30)	*χ* ^2^(1) = 2.80, *p* = .14
Cohabitating	Yes	72.2% (26)	73.5% (25)	*χ* ^2^(1) = 0.15, *p* = .99
Employed	Yes	80.6% (29)	67.6% (23)	*χ* ^2^(1) = 1.53, *p* = .28
Born in the Netherlands	Yes	91.7% (33)	91.4% (33)	*χ* ^2^(1) = 0.16, *p* = .99
Years nightmares	6 months to <1 year	5.6% (2)	2.9% (1)	*χ* ^2^(3) = 4.47, *p* = .22
	1 year to <5 years	11.1% (4)	17.6% (6)	
	5 years to <10 years	22.8% (8)	5.9% (2)	
	≥10 years	61.1% (22)	73.5% (25)	
Sleep medication	Yes	8.3% (3)	20.6% (7)	*χ* ^2^(1) = 1.61, *p* = .20
Anti‐depressants	Yes	13.9% (5)	11.8% (4)	*χ* ^2^(1) = 0.69, *p* > .99

Abbreviations: IRT, imagery rehearsal therapy; WL, wait‐list.

### 2.2 Power

The power for the current study was based on Kunze et al. (2017), who observed a between‐group effect size of *d *= 0.74. For the current study, we used a more conservative effect size estimate of Cohen's *d *= 0.60. Based on this effect size, groups of *n* = 30 were needed (power = 0.8; alpha = 0.05) to detect a significant difference at post‐test. In the original study protocol, we aimed to include a sample size of *n* = 100 to increase the power for the mediation analysis. However, due to practical issues recruitments was slower than expected. Therefore, we settled on including 70 participants.

### 2.3 Measurements

All measures were assessed at pre‐, post‐ and follow‐up tests (3 and 6 months). Additionally, primary measures and mediators were assessed weekly between pre‐ and post.

### 2.4 Primary measures

In line with Kunze et al. (2017), the primary outcome measures were nightmare frequency per week and nightmare distress. Nightmare frequency was measured with the Nightmare Frequency Questionnaire (NFQ; Krakow et al., 2002) and comprises the accumulated number of nightmares per week. The NFQ also measures the number of nights with nightmares per week, which was a secondary outcome in this study.

Nightmare distress was measured by the Nightmare Distress and Impact Questionnaire (NDIQ). This instrument was developed for an earlier study on the treatment of nightmares (Kunze et al., 2017). The questionnaire consists of two subscales, one measuring the daytime impact of nightmares and one measuring the discomfort caused by nightmares during the night. Both subscales consist of six items scored on a four‐point Likert scale (0—Not applicable to 3—Fully applicable). The questionnaire ranges from 0 to 36, with higher scores indicating more nightmare distress. This questionnaire appeared reliable in the earlier study (Cronbach's *α* = 0.75) and in the current study (0.67).

### 2.5 Secondary measures

Insomnia severity was measured with the Insomnia Severity Index (ISI; Bastien, Vallières, & Morin, 2001). The ISI is a seven‐item scale scored on a five‐point Likert scale. The range of the ISI is 0–28, with higher scores indicating more complaints. The ISI has good psychometric properties (Cronbach's *α* = 0.78). The Cronbach's *α* = 0.67 in the current sample.

Depressive complaints were measured with the Dutch version of a nine‐item depression scale of the Patient Health Questionnaire‐9 (PHQ‐9; range 0–27, Cronbach's *α* = 0.94; Spitzer, Kroenke, & Williams, 1999). The PHQ‐9 is scored on a five‐point Likert scale ranging from 0 (never) to 4 (almost daily), with higher scores indicating more complaints (range 0–36). The Cronbach's *α* = 0.82 in the current sample.

Anxiety symptoms were assessed with the Dutch version of the seven anxiety items of the Hospital Anxiety and Depression Scale (HADS‐A; Spinhoven et al., 1997; Zigmond & Snaith, 1983). The HADS‐A is scored on a four‐point Likert scale (0–3), with higher scores indicating more anxiety (range 0–21). The reliability of the HADS is good (*α* = 0.80–0.84), as is the test−retest correlation (*r* = .89; *p* < .001). The Cronbach's *α* = 0.75 in the current sample.

### 2.6 Mediation measure

Mastery was measured with a Visual Analogue Scale (VAS) from 0 (Totally disagree) to 100 (Totally agree). The statement used for the VAS was “I have control over the content of my nightmares”.

### 2.7 Measures to check for exclusion criteria

Post‐traumatic stress disorder was checked with the PSS. This is a valid and reliable (Cronbach's *α* = 0.85) self‐report questionnaire (Foa et al., 1993). The questionnaire consists of three subscales on intrusion, avoidance and arousal, and has a total of 17 items. The items are scored on a four‐point Likert scale ranging from 0 (Never) to 3 (Very much), with higher scores indicating more symptoms.

Suicidal ideation was assessed with five dichotomous (yes/no) items, based on the Mini International Neuropsychiatric Interview (MINI). Psychosis/schizophrenia was assessed with a single dichotomous (yes/no) question. Both sleep medication and medication for psychological complaints were measured through a single dichotomous (yes/no) question. If this was answered positively, questions followed about the type of medication and change in dosage in the last 6 weeks. Both alcohol and cannabis use were asked with a single question, whereby quantity could be indicated in different categories. Participants were excluded if they indicated either more than three or more glasses of alcohol a day for at least 21 days per month or more than once a week cannabis use.

### 2.8 Treatment

The treatment was based on the adapted IRT protocol used by Kunze et al. (2017), and more traditional IRT protocols (Krakow & Zadra, 2006). The treatment manual consisted of very few psycho‐educative elements, and mainly focused on practical information about how the treatment would be applied. The guided self‐help treatment protocol comprised three sessions with similar content and was sent to the participants in a pdf file. In session 1, participants were instructed to select the nightmare that they suffered from most frequently. Participants wrote down the original nightmare narrative and were asked to choose a moment in the nightmare when they wanted to change the storyline. The instructions were that this moment should be after the negative emotions were already activated; the most appropriate moment would typically be just before awakening from the nightmare. From that point onwards, participants were instructed to change their nightmare in “any way they wished”. After writing down the new storyline of the nightmare, they were asked to imagine this nightmare in the daytime. During this exercise, participants were instructed to imagine the original as vividly and emotionally as possible. In line with other rescripting‐based treatment protocols (Arntz & Weertman, 1999) and in order to refrain from prolonged exposure, we informed participants that they should directly move on to the rescripting part of the exercise as soon as the negative emotions associated with the nightmare were adequately activated. When the rescripting started, participants changed the nightmare in their imagination until emotions were subsided, and they were instructed to carry on with the rescripting of the nightmare until all needs were met. We informed the participants that the full exercises could take anywhere between a couple of minutes to 20–30 min. For homework, the participants were asked to do the same imagination exercise as described above. Sessions 2 and 3 had content similar to session 1. In session 2 there was some additional attention for troubleshooting (e.g. how to keep on doing the exercises), and in session 3 there was the option to work with a new nightmare.

After each session, participants filled out an online form where they reported on their experiences. Thereafter, participants were telephoned by undergraduate psychology students to help them with their imagination exercises. During the phone call they could ask questions that had arisen during the exercises. Furthermore, the undergraduate students helped with defining new nightmare scripts and motivated participants to keep on carrying out the exercises. If needed, the undergraduate students also helped with activating the nightmare memory. The coaches did so by advising them to focus on sensory information (e.g. next time you do the imagination exercises try to focus on what you see, hear, feel). The students had weekly supervision from the first two authors (JL and ME). The average phone‐call time was about 15 min per session, thus 45 min in total for the full treatment.

### 2.9 Procedure

Interested volunteers gave their informed consent online. Subsequently, participants filled out an online screener assessing the inclusion/exclusion criteria (see Figure 1 for a flowchart) and a baseline measurement of all outcomes (pre‐test/T0). Eligible participants were called by a research assistant who further explained the study, checked all inclusion/exclusion criteria, and assessed DSM‐5 nightmare disorder criteria. After this phone call a final decision was made about the inclusion of the participant. Included participants were then randomized to either IRT or a WL condition. If participants were randomized to the WL they were informed that they would receive IRT after filling out the post‐test. Randomization was performed by an independent researcher. The randomization order was generated using an online randomization tool with random blocks of two, four and six. From then on, weekly measurements were sent every Monday (T1–T5). Participants in the IRT condition started the treatment after filling out T1 (please see Figure S1 for a study overview). The treatment took 3 weeks. Measurements were carried on for one extra week to allow for any delay in the treatment. Post‐test (T5) was after 5 weeks. Participants in the IRT condition received follow‐up measurements 3 (T6) and 6 months (T7) after the post‐test. The study was approved by the internal ethical review board of the University of Amsterdam (2018‐CP‐8830), and was registered at www.trialregister.nl (NTR7077).

### 2.10 Statistical methods

Data integrity checks included valid values and range checks. In line with previous studies (Kunze et al., 2017; Lancee et al., 2010), nightmare frequency was log‐transformed to meet the normality assumption. For the post‐test effect, linear mixed (multilevel) regression analyses were conducted to evaluate the within‐group (Time) and between‐group (Time × Condition) effects of the intervention. The basic model was a two‐level (participants and measurement points) repeated‐measures design with the outcomes as dependent variable (i.e. nightmare frequency, nights with nightmares, mastery, NDIQ, PHQ, HADS‐A, ISI), Treatment as between‐subjects factor (IR versus WL), and Time as within‐subject factor (T0−T5 for nightmare frequency, nights with nightmares, mastery and NDIQ; pre‐ versus post‐assessment for PHQ, HADS‐A and ISI). Mixed regression analyses were based on the intention‐to‐treat principle (i.e. all randomized participants were included in the analyses). Effects were examined by modelling time effects using an unstructured covariance structure for the repeated‐part of the model, as being the best fitting model for the data.

Pre‐treatment differences on demographic and clinical variables between the two groups were explored. No pre‐treatment differences were observed on any variables. We also explored if any variable was related to non‐response on the post‐test. Chi‐square analyses showed that in the IRT condition, people of higher education more often completed the post‐test measure, *χ* ^2^(1) = 9.05; *p* < .01. To control for this, educational level was added to all analyses as a covariate (i.e. fixed effects; main effects, no interactions).

Cohen's *d* (Cohen, 1988) was used as an effect size, and was computed from the multilevel estimated means and observed standard deviations. Within‐condition change was defined as Δ*d* = (*M*_pre_ − *M*_post_)/*SD*_pooled‐pre_, where *SD*_pooled‐pre_ = √[(*SD*_preIR_^2^ + *SD*_preWL_^2^)/2]. Between‐group effect sizes were determined by calculating the difference between the within‐condition effect size; Δ*d*_between_ = [(*M*_preIR_‐*M*_postIR_) − (*M*_preWL_‐*M*_postWL_)]/*SD*_pooled‐pre_ (Morris, 2008).

All effects were tested at the 0.05 *α*‐level (two‐tailed). Analyses were carried out in SPSS version 24. Results are reported in accordance with the CONSORT guidelines for reporting clinical trials (Moher et al., 2012).

For the mediation analyses, we used a bootstrapping procedure that is implemented in Hayes’ SPSS PROCESS tool (Hayes, 2013). Bootstrapping is a non‐parametrical technique that generates an estimate of the sample based on several re‐samples, in this case *n* = 50,000. The mediation is tested by evaluating the 95% confidence interval of the indirect effect. In the mediation model, we added the independent variable (condition), the dependent variable (nightmare frequency/distress at post‐test—T5) and the mediator variable (mastery measured at time‐point three—T3). As covariates, we added the pre‐test—T0 levels of the dependent and the mediator variable as well as education level. We also calculated the proportion of effect of the independent variable that is accounted for by the mediator using 1 – *c*′/*c* (MacKinnon, Fairchild, & Fritz, 2007; Figure 3 illustrates the mediation model).

## 3 RESULTS

### 3.1 Completed sessions

In the IRT condition, 26 participants completed all sessions, two completed two sessions, seven completed one session, and one did not start the treatment after randomization.

### 3.2 Treatment outcomes

**FIGURE 2:**
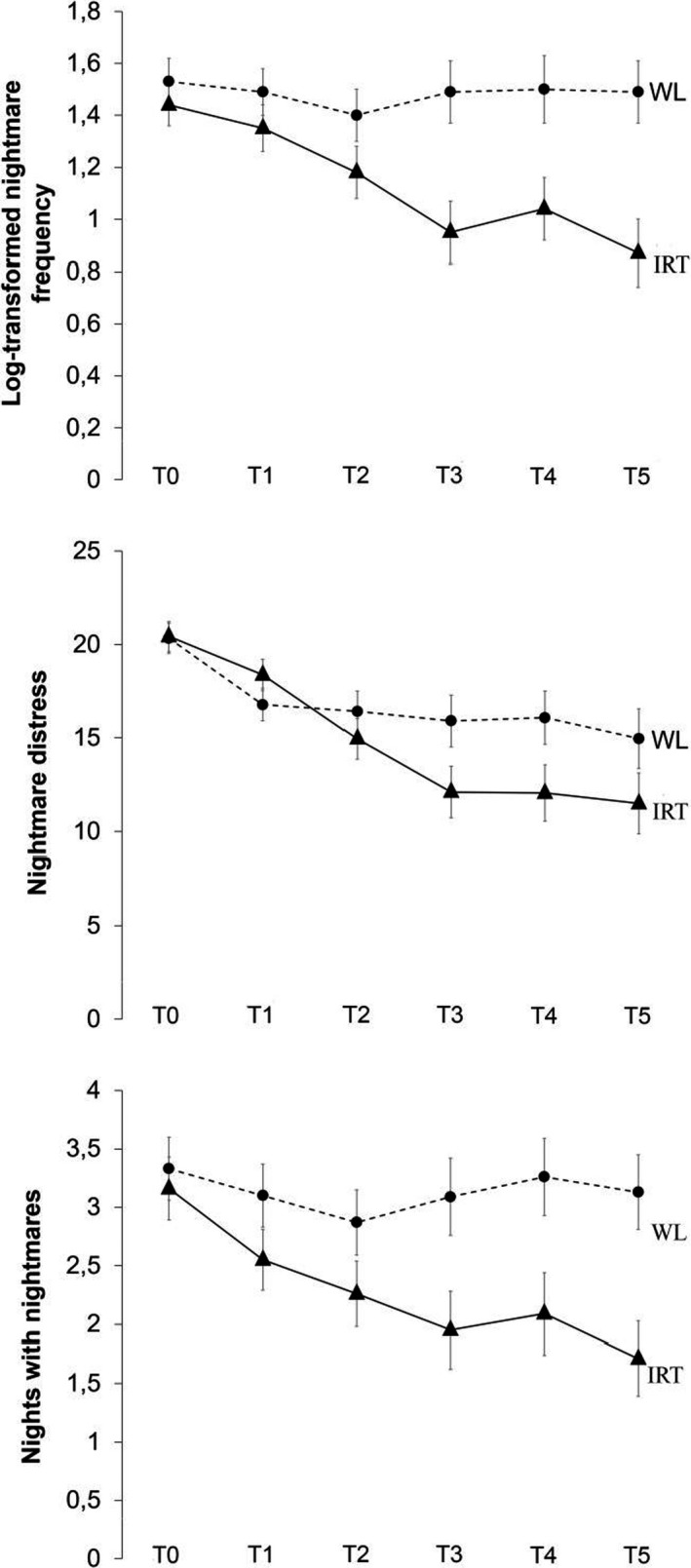
Log‐transformed nightmare frequency, nights with nightmares and nightmare distress over time. IRT, imagery rehearsal therapy; WL, wait‐list

Multilevel regression analyses based on all available time‐points revealed significant Treatment × Time interactions for nightmare frequency (*F*_5,63.13 _= 2.81, *p* = .024, *d* = 1.03), nightmare distress (NDIQ; *F*_5,62.30_ = 3.02, *p* = .017, *d* = 0.75), mastery (*F*_5,60.47_ = 9.43, *p* < .001, *d* = 1.77) and insomnia complaints (ISI; *F*_1,57.82_ = 20.60, *p* < .001, *d* = 1.12), indicating that IRT differed from WL over time (Figure 2; see Table 2 for corresponding estimated means and within‐ and between‐group effect sizes). Observed means and standard deviations of all outcome measures are depicted in Table S1. No significant Treatment × Time interactions were found for nights with nightmares (*F*_5,63.19_ = 1.62, *p* = .169, *d* = 0.76), depressive symptoms (PHQ; *F*_1,59.79_ = 1.68, *p* = .199, *d* = 0.30) and anxiety symptoms (HADS‐A; *F*_1,60.84_ = 0.49, *p* = .488, *d* = 0.19). The treatment effect for nights with nightmares did reach significance for the analysis based on the pre‐ and post‐test only (*F*_1,62.20_ = 5.97, *p* = .017, *d* = 0.71; leaving out T1–T4). The effects were sustained at 3‐ and 6‐months follow‐up (Tables 3, and S2 and S3).

**TABLE 2:** Corrected mixed‐regression based estimated means and standard errors (*SE*)

		Pre‐		Post‐		Cohen's *d*	
	Group	Mean	*SE*	Mean	*SE*	Within‐group	Between‐group
Log‐transformed	IRT	1.44	0.08	0.87	0.13	1.11	1.03*
Nightmare frequency	WL	1.53	0.09	1.49	0.12	0.08	
Nights with nightmares	IRT	3.16	0.27	1.71	0.32	0.88	0.76^ns^ ^a^
	WL	3.33	0.27	3.13	0.32	0.12	
Nightmare distress (NDIQ)	IRT	20.43	0.80	11.49	1.63	1.87	0.75*
	WL	20.33	0.81	14.96	1.59	1.12	
Mastery	IRT	11.86	3.18	51.47	4.82	−2.10	−1.77^***^
	WL	11.97	3.24	18.17	4.78	−0.33	
Depressive symptoms (PHQ)	IRT	8.98	0.79	6.64	0.79	0.50	0.30^ns^
	WL	9.29	0.81	8.33	0.77	0.21	
Anxiety (HADS‐A)	IRT	7.65	0.62	7.10	0.73	0.15	0.19^ns^
	WL	7.09	0.63	7.25	0.70	−0.04	
Insomnia severity (ISI)	IRT	15.09	0.72	10.45	0.88	1.07	1.12^***^
	WL	14.79	0.73	15.03	0.84	−0.06	

Note

*d*_within_ = (*M*_pre_ − *M*_post_)/*SD*_pooled‐pre_; *d*_between_ = [(*M*_preIR_ − *M*_postIR_) − (*M*_preWL_ − *M*_postWL_)]/*SD*_pooled‐pre_; means for effect size calculations were based on mixed‐regression based estimated means; *SD*s for effect size calculations were based on the observed values.

Abbreviations: HADS‐A, Hospital Anxiety and Depression Scale‐Anxiety; IRT, imagery rehearsal therapy; ISI, Insomnia Severity Index; NDIQ, Nightmare Distress and Impact Questionnaire; PHQ, Patient Health Questionnaire; WL, wait‐list.

^a^ Pre‐post analyses were significant (*F*_1,62.20_ = 5.97, *p* = .017, *d* = 0.71).

* *p* < .05; ^***^ *p* < .001.

**TABLE 3:** Corrected mixed‐regression based estimated means and standard errors (*SE*) of the follow‐ups for IRT

		3‐months follow‐up		6‐months follow‐up		Cohen's *d* relative to pre‐rest	
	Group	Mean	*SE*	Mean	*SE*	3 months	6 months
Nightmare frequency week	IRT	0.91	0.13	0.84	0.19	1.03	1.17
Nights with nightmares per week	IRT	1.66	0.27	1.72	0.43	0.91	0.88
Nightmare distress (NDIQ)	IRT	9.66	1.52	8.13	1.74	2.25	2.57
Mastery	IRT	52.16	5.81	60.76	6.32	2.14	2.59
Depression (PHQ)	IRT	6.30	1.03	4.64	0.67	0.47	0.56
Anxiety (HADS‐A)	IRT	6.13	0.70	4.18	0.72	0.42	0.95
Insomnia severity (ISI)	IRT	7.95	1.04	8.68	1.32	1.64	1.47

Note

*d* = (*M*_pre_ − *M*_post_)/*SD*_pooled‐pre_.

Abbreviations: HADS‐A, Hospital Anxiety and Depression Scale‐Anxiety; IRT, imagery rehearsal therapy; ISI, Insomnia Severity Index; NDIQ, Nightmare Distress and Impact Questionnaire; PHQ, Patient Health Questionnaire.

### 3.3 Mediation analysis

**FIGURE 3:**
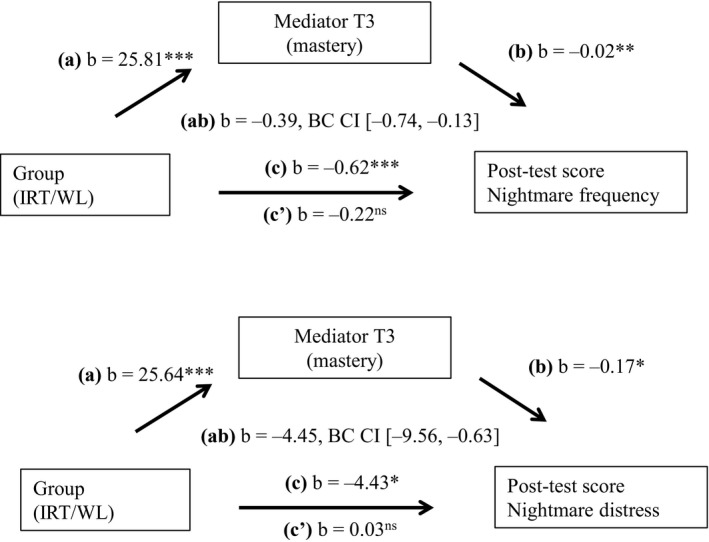
Mediation effects with mastery (T3) as mediator for nightmare frequency and distress (post‐test). Note: nightmare frequency is log‐transformed. In the model pre‐test scores of the mediator and the dependent variable as well as education level were added as covariates

We conducted mediation analyses for the dependent variables: “log‐transformed nightmare frequency” and “nightmare distress”. The mastery item assessed on a VAS at T3 was the mediator variable. As can be seen in Figure 3, mediation analyses showed that mastery mediates the treatment effects of IRT on nightmare frequency (*b* = −0.39, *BC CI* [−0.74, −0.13]), as well as on nightmare distress (*b* = −4.45, *BC CI* [−9.56, −0.63]; proportion explained, respectively, 45% and 41%; MacKinnon et al., 2007).

## 4 DISCUSSION

In this study, we investigated the efficacy of telephone‐guided IRT for nightmares. We observed that IRT more effectively reduced nightmare frequency, nightmare distress and insomnia severity than a WL condition. Even though the effects on nights with nightmares did not reach the statistical threshold (possibly due to power), all effect sizes were in the moderate to large range. These effects are in general larger than those observed in earlier meta‐analytic findings on the efficacy of mostly face‐to‐face IRT treatment (Augedal et al., 2013; Hansen et al., 2013; Seda et al., 2015; Yücel et al., 2020). The effects are similar to an earlier study using an isolated IRT format in a face‐to‐face setting (Kunze et al., 2017). Finally, the results are comparable to IRT in a self‐help format (Lancee et al., 2010) and online setting (Gieselmann et al., 2017). Overall, the present findings support the feasibility of guided self‐help IRT in the treatment of nightmares.

Another aim of this study was to replicate the finding that mastery mediates the effect of IRT for nightmares. In line with Kunze et al. (2019), we found that the current effects on both nightmare frequency and distress were mediated by mastery. This supports our earlier suggestion that mastery is an important concept and a probable mechanism within rescripting‐based treatments of nightmares (Kunze et al., 2019). Treatment development should therefore focus on targeting mastery. A possible way to do so may be a more explicit explanation of this concept within the treatment rationale (i.e. explaining *why* it is necessary to increase mastery). Another option may be to explicitly ask patients after their rescripting to indicate on which parts of the narrative they subjectively achieved more mastery, and to subsequently adjust the treatment plan to increase mastery in other parts of the narrative as well. In addition, Rousseau and Belleville (2018) argue that there may be confusion about the object over which mastery should be gained (e.g. the nightmare scenario, the general dream process, one's own imagery system, etc.). In the current study, we choose to focus on mastery over the nightmare scenario, but it may very well be that we tapped into (or missed) other relevant parts of the concept of mastery.

There were also limitations to this study. We excluded individuals with possible PTSD. This may have led to excluding the people that may need the treatment the most. The reason for excluding individuals with PTSD was that IRT was not yet tested for PTSD patients in a self‐help format. For safety reasons, we first wanted to evaluate the online treatment in a sample with few co‐morbidities. Now that it proved effective, we see no reason to refrain from further testing in a more clinical sample. This is further supported by an uncontrolled trial that recently reported promising findings of online‐delivered IRT for patients with PTSD (Putois et al., 2019).

Other limitations were that the sample consisted of predominantly white females of higher education, and that we did not use objective measurements for sleep. Another issue is the telephone‐guided feedback. In online treatment formats, text‐based feedback is routinely used. This is often more convenient as therapist and patient do not necessarily need to work on the exercises at the same time, which makes the treatment even more flexible. We decided against text‐based feedback, because this type of communication caused several misunderstandings in a pilot study, particularly regarding the imagination exercise and imagery rescripting of the nightmare. Related to this issue is our decision to give feedback *after* the exercises were carried out, whereas in face‐to‐face treatment feedback is given during the imagery rescripting. We were not sure if people would accept this type of direct intervention in the form of telephone feedback. In future studies, this could be tested with, for instance, video‐delivered feedback parallel to the imagery rescripting. Another limitation of this study was that we did not record the conversations between participants and coaches. This may be a missed opportunity as this could have helped our understanding on what type of feedback is most effective.

In the same vein, we recommend that text‐based feedback should undergo further testing, especially as Gieselmann et al. (2017) have successfully employed this procedure.

Taking these limitations into account, we argue that the results of this randomized controlled trial (RCT) are very promising. With about 45 min of therapist time for the full treatment, it is possible to deliver an effective telephone‐guided treatment for nightmares. The findings call for further investigation of online or self‐help IRT, starting with samples with post‐traumatic complaints. To increase the efficacy of (online) IRT, “mastery” seems to be an interesting candidate treatment target. At the same time, the efficacy of online IRT may also be enhanced by directly delivering feedback during the exercises through video calls. Additionally, media‐rich programmes (E.g., Espie et al., 2012) may further increase effects and limit treatment dropout in general. Whether these changes and/or additions to the current IRT protocol are indeed an improvement should be subject to empirical testing. The findings of this RCT warrant that time and effort is dedicated to these issues.

## CONFLICT OF INTEREST

No conflicts of interest declared.

## AUTHOR CONTRIBUTIONS

J. L., M. E. and A. E. K. contributed to the design of the study. J. L. and M. E. carried out recruitment and data collection, and supervised the treatment delivery. J. L. and A. E. K. performed the statistical analysis. J. L. drafted the manuscript. All authors contributed to and approved the paper.

## Supporting information

Supinfo
