# Supplementary material for: Telephone‐guided imagery rehearsal therapy for nightmares: Efficacy and mediator of change
Source: J Sleep Res. 2020 Jun 21;30(3):e13123. doi: 10.1111/jsr.13123 (PMC8244061; doi:10.1111/jsr.13123)
Supplement: Supplementary file 1 — Supinfo [file JSR-30-e13123-s001.docx]

**Supplemental Tables and Figure**

**Supplemental Figure S1**

**
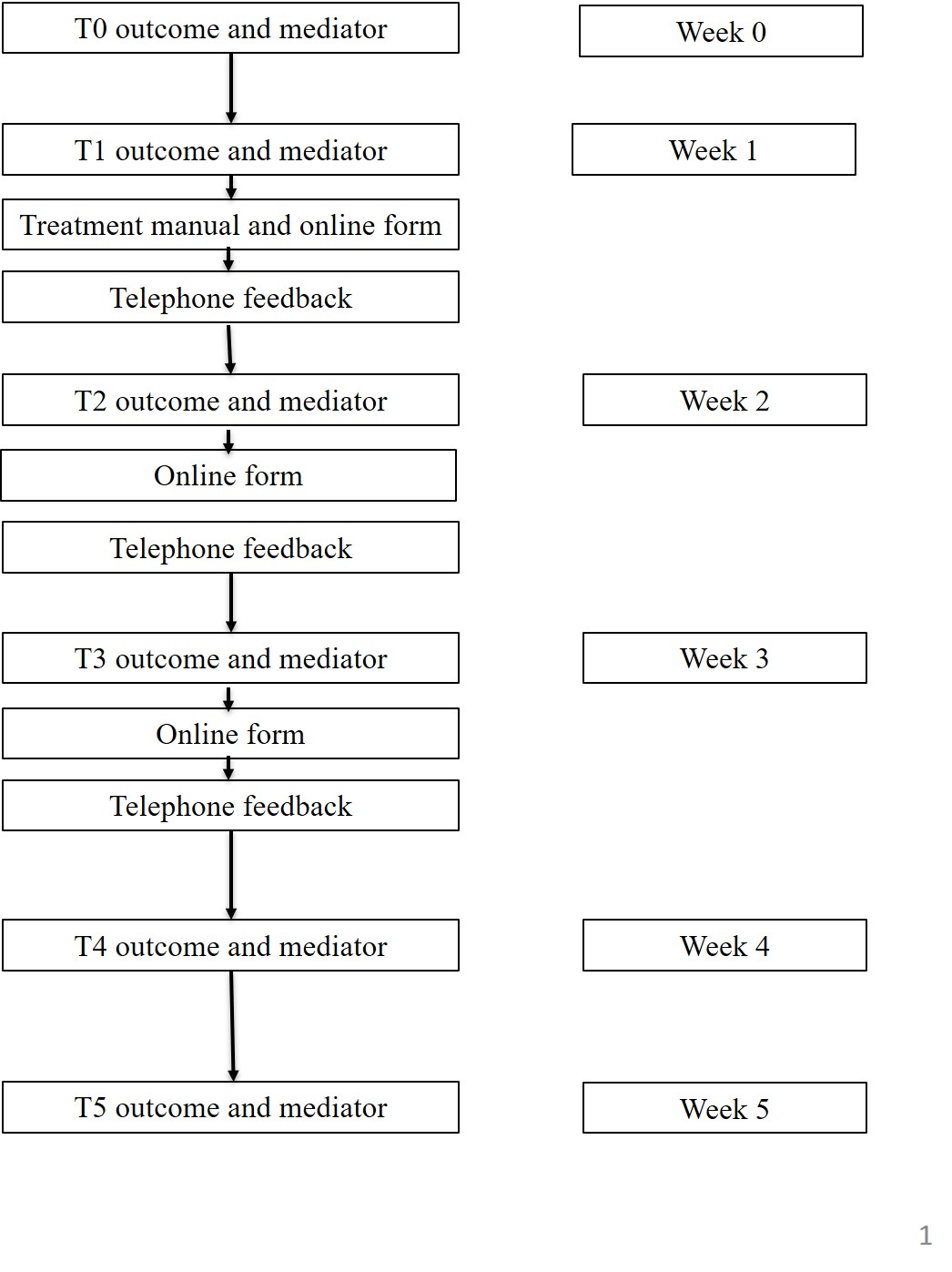
**

| **Supplemental Table 1**. Observed means and standard deviations (SD) | | | | | | | | | | |
| --- | --- | --- | --- | --- | --- | --- | --- | --- | --- | --- |
|  |  |  | Pre | |  | Post | |  | *Cohen's d* | |
|  | Group |  | Mean | SD |  | Mean | SD |  | Within group | Between group |
| NFweek | IRT |  | 3.67 | 2.12 |  | 1.73 | 1.64 |  | 0.60 | 0.58 |
|  | WL |  | 4.56 | 4.08 |  | 4.52 | 3.56 |  | 0.01 |  |
| NFnight | IRT |  | 3.22 | 1.51 |  | 1.70 | 1.66 |  | 0.92 | 0.78 |
|  | WL |  | 3.29 | 1.77 |  | 3.06 | 2.02 |  | 0.14 |  |
| NDIQ | IRT |  | 20.58 | 5.07 |  | 10.15 | 9.12 |  | 2.18 | 0.98 |
|  | WL |  | 20.24 | 4.48 |  | 14.48 | 8.15 |  | 1.20 |  |
| Mastery | IRT |  | 12.40 | 16.88 |  | 50.22 | 32.63 |  | 2.01 | 1.65 |
|  | WL |  | 11.30 | 20.64 |  | 17.97 | 22.77 |  | 0.35 |  |
| PHQ | IRT |  | 8.89 | 4.37 |  | 6.59 | 4.43 |  | 0.49 | 0.27 |
|  | WL |  | 9.22 | 4.94 |  | 8.20 | 4.36 |  | 0.22 |  |
| HADS-A | IRT |  | 7.43 | 3.58 |  | 7.11 | 4.29 |  | 0.09 | 0.10 |
|  | WL |  | 7.15 | 3.73 |  | 7.19 | 3.94 |  | 0.01 |  |
| ISI | IRT |  | 15.26 | 4.30 |  | 10.70 | 4.89 |  | 1.05 | 1.02 |
|  | WL |  | 14.76 | 4.40 |  | 14.65 | 4.61 |  | 0.03 |  |
| Note: *d*_within_ = (*M*_pre_ - *M_post_*)/*SD*_pooled-pre_; *d*_between_ = [(*M*_preIR_-*M*_postIR_)-(*M*_preWL_-*M*_postWL_)]/*SD*_pooled-pre_ | | | | | | | | | | |
|  | | | | | | | | | | |

| **Supplemental Table 2**. Observed means and standard deviations (SD) of the follow-ups for IRT | | | | | | | | | |
| --- | --- | --- | --- | --- | --- | --- | --- | --- | --- |
|  |  | 3-months FU | |  | 6-month FU | |  | *Cohen's d* | |
|  |  | Mean | SD |  | Mean | SD |  | 3-months | 6-months |
| Nightmare frequency week |  | 2.04 | 2.05 |  | 2.25 | 3.68 |  | 0.50 | 0.44 |
| Nights with nightmares per week |  | 1.64 | 1.38 |  | 1.50 | 1.75 |  | 0.96 | 1.05 |
| Nightmare distress (NDIQ) |  | 9.72 | 7.94 |  | 8.00 | 7.15 |  | 2.27 | 2.63 |
| Mastery |  | 52.32 | 29.89 |  | 59.13 | 26.91 |  | 2.12 | 2.48 |
| Depression (PHQ) |  | 5.68 | 5.42 |  | 4.13 | 3.32 |  | 0.69 | 1.02 |
| Anxiety (HADS-A) |  | 5.84 | 3.60 |  | 4.00 | 3.08 |  | 0.43 | 0.94 |
| Insomnia severity (ISI) |  | 7.72 | 5.44 |  | 8.69 | 5.46 |  | 1.73 | 1.51 |
| Note: *d* = (*M*_pre_ - *M_post_*)/*SD*_pooled-pre_ | | | | | | | | | |

| **Supplemental Table 3**. Multilevel regression estimates for the time effects of IRT on the 3- and 6-month follow-up relative to the pre-test | | | |
| --- | --- | --- | --- |
|  |  | 3-month | 6-month |
| Nightmare frequency week |  | *F*(1, 25.08) = 16.14, *p* < .001 | *F*(1, 16.57) = 9.95, *p* < .01 |
| Nights with nightmares per week |  | *F*(1, 26.45) = 20.28, *p* < .001 | *F*(1, 16.02) = 13.25, *p* < .01 |
| Nightmare distress (NDIQ) |  | *F*(1, 25.21) = 60.36, *p* < .001 | *F*(1, 18.11) = 45.00, *p* < .001 |
| Mastery |  | *F*(1, 27.91) = 40.29, *p* < .001 | *F*(1, 16.42) = 60.96, *p* < .001 |
| Depression (PHQ) |  | *F*(1, 25.17) = 9.45, *p* < .01 | *F*(1, 18.10) = 21.61, *p* < .001 |
| Anxiety (HADS-A) |  | *F*(1, 25.04) = 3.35, *p* = .08 | *F*(1, 18.10) = 14.98, *p* < .01 |
| Insomnia severity (ISI) |  | *F*(1, 28.49) = 45.48, *p* < .001 | *F*(1, 18.30) = 22.34, *p* < .001 |
